# Awareness of Head and Neck Cancers: A 2021 Nationwide Cross-Sectional Survey in Poland

**DOI:** 10.3390/jcm11030538

**Published:** 2022-01-21

**Authors:** Wojciech Pinkas, Mateusz Jankowski, Waldemar Wierzba

**Affiliations:** 1 Central Clinical Hospital of the Ministry of the Interior and Administration in Warsaw, 02-507 Warsaw, Poland; waldemar.wierzba@cskmswia.gov.pl; 2 UHE Satellite Campus in Warsaw, University of Humanities and Economics in Łódź, 01-513 Warsaw, Poland; 3 School of Public Health, Centre of Postgraduate Medical Education, 01-826 Warsaw, Poland; mjankowski@cmkp.edu.pl

**Keywords:** head and neck cancers, general population’s knowledge, risk factors, cancer, Poland

## Abstract

Head and neck cancers (HNC) are the seventh most common cancers worldwide. Early diagnosis of HNC is associated with better outcomes. This study aimed to assess public awareness of HNC among adults in Poland, with particular emphasis on awareness of HNC symptoms and risk factors for HNC. This cross-sectional study was carried out in November 2021 on a nationwide, representative sample of 1082 inhabitants of Poland aged 18 years and over. The computer-assisted web interview (CAWI) technique was used. Most of the respondents rated their knowledge of head and neck cancers as “a little” (40.8%) or “nothing at all” (30%). The most identified symptoms were a lump in the neck (57.9%) and swelling or a lump in the throat (51.8%). The most identified risk factor for HNC was smoking cigarettes/tobacco (63.1%). Excessive alcohol consumption and HPV infection were correctly identified as risk factors by about one-third of respondents. Public awareness of selected symptoms of HNC and risk factors for HNC differed by sociodemographic factors, of which the educational level was the most important factor associated with awareness of head and neck cancers (*p* < 0.05). This study demonstrated low public awareness of head and neck cancers among adults in Poland.

## 1. Introduction

Head and neck cancers (HNC) are still a current and important problem in daily otolaryngological practice [1,2]. Approximately 600,000 new HNC cases are diagnosed in the world every year [3,4,5]. In 2018, head and neck cancers were the seventh most common cancer worldwide [1]. It is estimated that HNC accounts for approximately 6% of all cancer cases and approximately 5% of cancer-related deaths worldwide [3,4,5]. 

Head and neck cancer is 2–4 times more common in males [6]. The risk of developing HNC increases with age, especially after 50 years of age [1,6]. Head and neck cancers are strongly associated with certain environmental and lifestyle risk factors like tobacco smoke and alcohol consumption [7,8]. It is estimated that up to 80% of head and neck cancers are linked to tobacco use [7]. Moreover, in recent years, the impact of certain types of human papillomavirus (HPV) on the development of HNC has been thoroughly documented [9]. This virus is detected in young patients not exposed to the major risk factors such as tobacco and alcohol [10]. It is estimated that from 60% to 70% of cancers in the oropharynx are linked to HPV infection [11,12]. 

The most common symptoms of head and neck cancers are a lump in the neck, persistent stinging or limited mobility of the tongue, persistent sore throat, chronic hoarseness, dysphagia and odynophagia [1,2,3,4,5,11]. Moreover, a disturbing symptom is the presence of tumors in the head and neck area, especially non-healing ulcers, erythro- and/or leukoplakia, epistaxis or unilateral nasal blockage [1,2,3,4,5]. Despite the intensified efforts of the research community to improve the quality of treatment, head and neck cancers are still characterized by a low five-year survival rate (approximately 45–60%, depending on the location of primary tumor and grade) [11,13]. HPV-associated HNC cancers are characterized by a younger age at onset, basaloid or warty histopathology and better prognosis when compared with their HPV-negative counterparts [12]. HNC often leads to severe disability and reduces the quality of life in patients who have completed the complicated diagnostic and therapeutic process [14,15]. 

Poland is a country with a high HNC burden compared to other European Union (EU) member-states [16,17]. The HNC incidence rate in Poland is approximately 1.4 times higher for men and 1.2 for women compared to the average for other EU countries [17]. Every year, about 5000 new HNC cases are reported in Poland [17]. Such a high HNC burden in Poland may result from the higher prevalence of tobacco and alcohol consumption compared to Western European countries [18,19].

Early diagnosis of HNC is associated with better outcomes. So, public awareness of symptoms of head and neck cancers is necessary to reduce the head and neck cancer burden. Moreover, awareness of risk factors for head and neck cancers is the major HNC prevention method. To raise awareness of tumors affecting areas between the head and the neck, cyclical educational campaigns on head and neck cancers are conducted (e.g., European Head and Neck Cancer Awareness Week) [20]. However, numerous studies showed that the public awareness of head and neck cancers is still relatively low [21,22,23]. Regular monitoring of public awareness of head and neck cancers is necessary to provide effective educational and preventive activities. Even though Poland is one of the countries with the highest head and neck cancer burden, there is a lack of nationally representative research assessing public awareness of HNC. Previously published data on awareness of HNC in Poland were limited to young adults (18–35 years) [24].

Therefore, this study aimed to assess public awareness of head and neck cancers among adults in Poland, with a particular emphasis on awareness of HNC symptoms and HNC risk factors.

## 2. Materials and Methods

### 2.1. Study Design and Population

This cross-sectional study was carried out between 5 and 8 November 2021 on a nationwide, representative sample of 1082 inhabitants of Poland aged 18 years and over. The computer-assisted web interview (CAWI) technique was used [25]. All the interviews were carried out by a specialized survey company (Ariadna online panel) [26] on behalf of the research team. 

Respondents were selected from the Ariadna online panel as a part of the Omnibus survey [26]. The operational number of the Ariadna online panel is over 110,000 registered and verified individuals aged 15 and over. The panel is actively updated to be representative of the adult Polish population. Data collection through the Ariadna online survey methodology (using a dedicated IT system) has been used in previously published papers [27,28,29].

Non-probability quota sampling was applied [26]. Respondents were selected based on the stratification model, including the factors of gender, age, size of domicile and territorial distribution within the 16 administrative regions (voivodeships) in Poland. The stratification was based on demographic data from the Central Statistical Office (CBOS, Warsaw, Poland) [30].

Participation in the study was voluntary and anonymous. All participants provided their informed consent. The study protocol was approved by the Ethics Review Board at the Central Clinical Hospital of the Ministry of the Interior and Administration in Warsaw, Poland (approval number 131/2021).

### 2.2. Questionnaire and Study Measures 

The research tool was a questionnaire developed for the purpose of this study. In preparation for the questionnaire, we analyzed the previously published nationwide cross-sectional surveys on head and neck cancers, with particular emphasis on the studies by Luryi et al. and Torabi et al. [21,22]. The questionnaire included nine questions related to the awareness of head and neck cancers. Questions also addressed personal characteristics.

At the beginning of the questionnaire, the following sentence was addressed to all the participants as an introduction: “Head and neck cancers are cancers that occur around the head and neck (outside of the brain and eyeball) and affect parts of the body such as the lips, mouth, throat, sinuses, salivary glands, larynx and ear.”

To determine the self-reported level of knowledge about head and neck cancers, respondents were asked to self-rate their HNC knowledge using the question: “How knowledgeable are you about head and neck cancers?”, with their answers given on a five-point Likert scale including not at all, a little, moderate, somewhat strong and very strong. 

Awareness of the symptoms of head and neck cancers: Respondents were asked about their awareness of the symptoms of head and neck cancers, using the question: “What do you think are the symptoms of head and neck cancers?”. Please select all that apply” with 15 mutually nonexclusive answers. Respondents were asked to select “yes” or “no” for each answer choice. 

Then, to determine the self-reported level of awareness of the risk factors for head and neck cancer, respondents were asked the question: “What do you think are the risk factors for head and neck cancer?”, with 10 mutually nonexclusive answer choices offered. Respondents were asked to select “yes” or “no” for each answer choice. 

Respondents were asked about their awareness of nationwide head and neck cancer prevention programs (e.g., European Head and Neck Cancer Awareness Week, the Polish Programme of Head and Neck Cancer Prevention and the “Don’t lose your head!” program of prevention and early detection of head and neck cancers). Moreover, respondents were asked about the type of doctors that they would go to in the first place if they suspected a head and neck cancer. A series of questions about HPV and its role in the development of head and neck cancers were also asked.

### 2.3. Statistical Analysis

The data were analyzed with SPSS version 28 (IBM, Armonk, NY, USA). The distribution of categorical variables was shown by frequencies and proportions. Statistical testing to compare categorical variables was completed using the independent samples chi-squared test.

Associations between sociodemographic factors (gender, age, marital status, children, place of residence, educational level, occupational status, self-reported financial situation) and awareness of selected symptoms of head and neck cancers were analyzed using logistic regression analyses. A lump in the neck, swelling or lump in the throat, chronic hoarseness, numbness of the tongue, mouth or lip and blocked nose on one side were considered separately as dependent variables in the model. These variables were selected because they are the most common symptoms of head and neck cancers that are well-described in the literature. 

Moreover, similar logistic regression analyses were carried out for the associations between sociodemographic factors and awareness of risk factors for head and neck cancers. The five best-documented risk factors for head and neck cancer—(1) excessive alcohol consumption, (2) smoking cigarettes/tobacco, (3) HPV infection, (4) excessive sunbathing and a (5) diet low in fruits—were considered separately as dependent variables in the model. 

In univariate logistic regression analyses, all variables were considered separately. Multivariate logistic regression analyses included all sociodemographic variables. The strength of association was measured by the odds ratio (OR) and 95% confidence intervals (CI). Statistical inference was based on the criterion *p* < 0.05.

## 3. Results

### 3.1. Characteristics of the Study Population

The analysis was based on responses to survey forms received from 1082 individuals (52.6% females), with a mean age of 44.5 ± SD (18–86 years). Almost one-third of the respondents lived in rural areas. Half of the respondents were married. Almost two-thirds of respondents were occupationally active. Among the respondents, 42.6% had higher education. The characteristics of the study population are presented in Table 1.

### 3.2. Awareness of Head and Neck Cancers 

Most of the respondents rated their knowledge about head and neck cancers as “a little” (40.8%) or “nothing at all” (30%). Over a quarter of the respondents (26.3%) self-rated their knowledge about head and neck cancers as moderate. Less than 4% of respondents declared somewhat strong (3.1%) or very strong (0.8%) knowledge about head and neck cancers. There were no significant differences in the self-reported level of knowledge about HNC by gender, age, educational level or place of residence. 

Among the respondents, 11.5% had ever heard about the European Head and Neck Cancer Awareness Week, the Polish Programme of Head and Neck Cancer Prevention or the “Don’t lose your head!” program of prevention and early detection of head and neck cancers. There were no significant differences in the percentage of respondents who were aware of the abovementioned head and neck cancer prevention programs by sociodemographic factors. 

Most of the respondents (61.8%) declared that if they suspected head and neck cancer, they would first go to their family doctor. Moreover, 22.2% of respondents indicated the oncologist, 7.6% laryngologist or 1% dentist as the first-choice doctor in case of suspected head and neck cancer. Among the respondents, 7.4% declared that they do not know which doctor they would see if they suspected head and neck cancer.

### 3.3. Respondents’ Knowledge Regarding the Symptoms of HNC and Risk Factors for HNC 

The most identified symptoms were a lump in the neck (57.9%) and swelling or a lump in the throat (51.8%). The least recognized symptom was a blocked nose on one side (12.7%). Almost 40% of the respondents identified pain or trouble swallowing (39.3%), bleeding in the mouth or throat (37.9%) and chronic hoarseness (37.7%) as symptoms of head and neck cancers. Slightly more than one-third of the respondents indicated numbness of the tongue, mouth or lip as a symptom of head and neck cancers. Among the respondents, 29.2% believed that red or white sores in the oral cavity that do not heal are symptoms of head and neck cancers. One-quarter of the respondents indicated ear pain as a symptom of head and neck cancers. Almost half of the respondents incorrectly believed that a headache may be a symptom of head and neck cancers. Moreover, among the respondents, 45.4% incorrectly believed that dizziness may be a symptom of head and neck cancers (with significant differences by gender). Further details are presented in Table 2.

The most identified risk factor for HNC was smoking cigarettes/tobacco (63.1%). Excessive alcohol consumption and HPV infection were correctly identified as risk factors by about one-third of respondents. The least recognized symptoms were excessive sunbathing (28%) and a diet low in fruits (15.6%). Among the respondents, 25.9% incorrectly believed that tooth decay is a risk factor for head and neck cancers. Moreover, 18.3% incorrectly declared that smoking marijuana can lead to head and neck cancer development. About one-tenth of respondents incorrectly believed that biting your cheeks, eating spicy or fatty foods or frequently talking on the cell phone are risk factors for head and neck cancers (Table 2). 

Females compared to males more often indicated a lump in the neck (61.9% vs. 53.4%; *p* = 0.005), swelling or lump in the throat (56.6% vs. 46.6%; *p* = 0.001), pain or trouble swallowing (42.4% vs. 35.9%; *p* = 0.03), chronic hoarseness (43.6% vs. 31.2%; *p* < 0.001), numbness of the tongue, mouth or lip (38.8% vs. 29.6%; *p* = 0.001) and persistent stinging of the tongue (21.4% vs. 15.2%; *p* = 0.01) as symptoms of head and neck cancers (Table 3). The proportion of respondents who indicated chronic hoarseness as a symptom of head and neck cancers increased with age (*p* < 0.001). Meanwhile, the proportion of respondents who indicated nasal bleeding as a symptom of head and neck cancers decreased with age (*p* = 0.03). Moreover, the proportion of respondents who indicated a lump in the neck, chronic hoarseness, nasal bleeding, and blocked nose on one side as symptoms of head and neck cancers differed by marital status (Table 3). Respondents who had children more often indicated chronic hoarseness as a symptom of head and neck cancers compared to those who did not have children (41.3% vs. 31.2%; *p* < 0.001). Further to this, the proportion of respondents who indicated persistent stinging of the tongue as a symptom of head and neck cancers differed by place of residence. The proportion of respondents who indicated a lump in the neck, swelling or lump in the throat, chronic hoarseness, ear pain, persistent stinging of the tongue or blocked nose on one side as symptoms of head and neck cancers was the highest among those respondents who had higher education (Table 3). There were no statistically significant differences in the respondents’ knowledge of the symptoms of head and neck cancers by self-reported financial situation. Further details are presented in Table 3. 

Respondents with higher education more often indicated excessive alcohol consumption, smoking cigarettes/tobacco, HPV infection and excessive sunbathing as risk factors for HNC compared to respondents who did not have higher education (Table 4). The proportion of respondents who indicated smoking cigarettes/tobacco and excessive sunbathing as risk factors for HNC differed by marital status (Table 4). There were no statistically significant differences in the respondents’ knowledge of the risk factors for HNC by gender, age, place of residence, children, occupational status or the self-reported financial situation. Further details are presented in Table 4.

### 3.4. Factors Associated with Awareness of Head and Neck Cancers 

The results of the univariate and multivariate regression analyses are presented in Table 5 and Table 6. When adjusted for all covariables, females had higher odds of indicating that a lump in the neck is a symptom of head and neck cancers (OR: 1.43, 95%CI: 1.11–1.83; *p* < 0.01). Females (OR: 1.56, 95%CI: 1.22–2.00; *p* < 0.01), respondents who had never been married (OR: 1.59, 95%CI: 1.11–2.27; *p* < 0.05) and those who had higher education (OR: 1.57, 95%CI: 1.22–2.03; *p* < 0.001) had higher odds of indicating that swelling or a lump in the throat is a symptom of head and neck cancer. Respondents aged 35–49 had lower odds of indicating that swelling or a lump in the throat is a symptom of head and neck cancer (OR: 0.59; 95%CI: 0.37–0.96; *p* < 0.05). Moreover, females (OR: 1.66, 95%CI: 1.28–2.14; *p* < 0.001) and respondents who had higher education (OR: 1.42, 95%CI: 1.09–1.84; *p* < 0.01) had higher odds of indicating that chronic hoarseness is a symptom of head and neck cancer. Those respondents who lived in cities with from 20,000 to 99,999 residents had lower odds of indicating that chronic hoarseness is a symptom of head and neck cancer compared to those who lived in cities with over 500,000 residents (OR: 0.66, 95%CI: 0.43–0.99; *p* < 0.05). Females (OR: 1.46, 95%CI: 1.13–1.90; *p* < 0.01) had higher odds of indicating that numbness of the tongue, mouth or lip is a symptom of head and neck cancer. Further details are presented in Table 5.

Respondents who had never been married (OR: 1.57, 95%CI: 1.09–2.26; *p* < 0.05), those who had higher education (OR: 1.33, 95%CI: 1.02–1.73; *p* < 0.05) and those with a rather good, good or very good financial situation (OR: 1.60, 95%CI: 1.10–2.33; *p* < 0.05) had higher odds of being aware that excessive alcohol consumption is a risk factor for head and neck cancers (Table 5). Respondents who had higher education had higher odds of being aware that smoking cigarettes/tobacco (OR: 1.32, 95%CI: 1.01–1.71; *p* < 0.05) and HPV infection (OR: 1.32, 95%CI: 1.01–1.73; *p* < 0.05) are risk factors for head and neck cancers. Moreover, respondents who self-declared a rather good, good or very good financial situation had higher odds of being aware that excessive sunbathing is a risk factor for head and neck cancers (OR: 1.49, 95%CI: 1.01–2.20; *p* < 0.05). Respondents aged 35–49 years had lower odds (OR: 0.44, 95%CI: 0.24–0.84; *p* < 0.05) of being aware that a diet low in fruits is a risk factor for head and neck cancers. Details are presented in Table 6.

## 4. Discussion

To the authors’ best knowledge, this is the first study on the public awareness of head and neck cancers in a representative sample of adults in Poland. This study revealed a low level of public awareness of head and neck cancers. More than 70% of respondents declared a lack of knowledge or little knowledge about head and neck cancers. Out of 12 symptoms of HNC, only two (lump in the neck and swelling or lump in the throat) were correctly indicated by more than half of the respondents. At the same time, almost half of the respondents incorrectly indicated a headache and dizziness as symptoms of HNC. The most identified risk factor for HNC was smoking cigarettes/tobacco and only one-third of respondents were aware that excessive alcohol consumption and HPV infection may lead to HNC. Public awareness of selected symptoms of HNC and risk factors for HNC differed by sociodemographic factors, of which the educational level was the most important factor associated with awareness of head and neck cancers. 

Data on public awareness of HNC are very limited. Two waves of a cross-sectional survey on public awareness of HNC in the US showed that between 2013 and 2020, the proportion of US inhabitants that indicated they were ‘‘not at all’’ or ‘‘not very’’ knowledgeable of HNC decreased from 66.1% in 2013 to 40% in 2020 [21]. The only available study on attitudes relating to HNC in Poland was carried out between 2014 and 2015 by Krentowska et al. [24]. In a group of students aged 18–35 years, 14.8% had never heard of HNC [24]. In this study, more than two-thirds of adult Poles declared a lack of knowledge or little knowledge of HNC. This percentage was higher than that previously reported by Krentowska et al. [24] or the percentage observed in the US by Torabi et al. [21]. 

Awareness of symptoms of HNC is critical to early detection of the disease. However, this study showed low awareness of major symptoms of HNC. In this study, a lump in the neck and swelling or lump in the throat were the most recognized symptoms of HNC, indicated by more than 50% of respondents. Similar findings were observed among young adults in Poland between 2014 and 2015 [24]. Krentowska et al. reported that slightly more than 50% of young adults in Poland (18–35 years) specified a tumor in the neck, swallowing problems, long-lasting hoarseness or a sore throat as HNC symptoms [24]. We can hypothesize that these two symptoms were the most recognized due to their impacts on daily life. The neck is a visible part of the body and a lump on the neck, for aesthetic reasons, may be easily noticeable. Moreover, a swelling or lump in the throat may disturb everyday functioning, including breathing, speaking and eating. The awareness of HNC symptoms presented in this study was much lower than in the US general adult population, where over 50% of respondents correctly indicated six different symptoms of HNC [21]. In the study by Krentowska et al., almost half of the respondents were medical students [24]. Krentowska et al. reported that the level of awareness of HNC was lower among non-medical students compared to medical students [24]. We can hypothesize that not only higher education but also a medical education background may have a significant impact on awareness of head and neck cancers.

Furthermore, in the case of most of the symptoms of HNC presented in this study, females and those with higher education were more aware of symptoms of HNC compared to males and those without higher education. We can hypothesize that females are more aware of HNC symptoms due to the gender gaps in health-related awareness [31]. Education is a fundamental social determinant of health [32]. Our findings showed that the educational level is also associated with the awareness of HNC symptoms. 

It is estimated that 21% of Poles aged 15 and over are daily smokers [33] and nearly 600,000–700,000 are dependent on alcohol [34]. In this study, smoking cigarettes/tobacco was the most recognized (63.1%) risk factor for HNC. In the study by Krentowska et al., tobacco use was also the most recognized risk factor for HNC, declared by 89.6% of young adults in Poland. High awareness of tobacco use as a risk factor for HNC may result from extensive anti-tobacco campaigns that were carried out in Poland in recent years [35]. Moreover, in Poland, pictorial health warnings on tobacco products (including those on HNC) are mandatory. Excessive alcohol consumption was indicated only by one-third of respondents. Such a low awareness of alcohol as a risk factor for HNC may result from the relatively low number of educational campaigns on the carcinogenic potential of alcohol consumption carried out by public health institutions in Poland.

Poland is a country with a low HPV vaccination coverage rate [36]. Since January 2021, HPV vaccines have been partially refunded as a part of the National Cancer Strategy [37]. HPV infection is one of the major risk factors of HNC. However, in this study, only one-third of respondents indicated HPV infection as a risk factor for HNC. Similar results were reported by Krentowska et al. [24]. Among young adults in Poland, over 62% were unaware that HNC may be associated with exposure to HPV [24]. Jeruzal-Świątecka et al. also reported that approximately half of the students in Poland were unaware of the link between HPV infection and the risk of HNC [38]. Most educational campaigns on HPV-related cancers are focused on cervical cancer. Our findings indicate that education on HPV infection and the risk of HNC should be considered as a part of cancer education programs. 

In this study, over 60% of the respondents would see the family doctor if they suspected head and neck cancer. Only 8% of respondents indicated laryngologists as their first-choice physician. This proves how important the role of family doctors is in HNC prophylaxis and early diagnosis as they tend to be the first medical professional that patients visit. Educational campaigns that improve awareness of risk factors and symptoms of head and neck cancer among healthcare professionals (especially family doctors) should be implemented. 

This study proved that educational campaigns led by the government are highly recommendable for predefined, high-risk, targeted populations [39]. This can enable early detection, and therefore, improve morbidity and mortality. In recent years in Poland, initiatives such as the “National Program of Primary Prevention and Early Detection of Head and Neck Neoplasms”, implemented by the Ministry of Health [20], and the “Do not lose your head” program of prevention and early cancer detection have been introduced [40]. From 2009 to 2020, the Polish Agency for Health Technology Assessment and Tariff System approved a total of 307 projects for preventive healthcare programs in the field of oncology. Only two of them concerned head and neck cancer, compared to 83 concerning breast cancer and 76 concerning prostate cancer [41]. Further preventive programs are needed to reduce the head and neck cancer burden in Poland.

This study has practical implications for cancer control policies. First, our findings suggest that there is an urgent need to conduct a nationwide information campaign on the symptoms of HNC and risk factors for HNC. Secondly, significant differences in the awareness of head and neck cancers by educational level point to the need to implement personalized communications targeted to particular social groups. Thirdly, our findings indicate a further need for disseminating knowledge about the path of a patient with suspected head and neck cancer and the role of laryngologists in the diagnosis and treatment of HNC. 

This study has several limitations. First, this study was carried out using the computer-assisted web interviewing (CAWI) research method. CAWI excludes the possibility of interaction with the respondent (and hence, the ability to assess the competencies of the respondents, i.e., whether they sufficiently understand the questions asked). Moreover, this research method includes only subjects who have internet access (though more than 90% of households in Poland now have internet access) [42]. Second, we cannot exclude non-response bias. Third, the use of closed-ended questions may suggest answers to the respondent. In this case, there were other risk factors for HNC—e.g., an immunocompromised patient or patient who has undergone an organ transplant, male gender and age >50—which were not included in this study. Nevertheless, the research tool and methods used in this study were comparable to previously published papers aimed at a similar research topic [21,24].

## 5. Conclusions

This study demonstrated low public awareness of head and neck cancers among adults in Poland. The educational level was the most important factor significantly associated with the awareness of the selected symptoms of head and neck cancers and risk factors for head and neck cancers. Moreover, the current study indicated significant gaps in the recognition of head and neck cancer prevention programs among adults in Poland. The presented data underscore the importance of adopting a cancer control strategy based on education, prevention and early detection of head and neck cancers.

## Figures and Tables

**Table 1:** Characteristics of the study population (*n* = 1082).

Variable	Total Sample *n* = 1082	
Overall	*n*	%
Gender		
Female	569	52.6
Male	513	47.4
Age (years)		
18–34	350	32.3
35–49	286	26.4
50–64	287	26.5
65+	159	14.7
Marital status		
single	236	21.8
married	546	50.5
informal relationship	161	14.9
divorced	65	6.0
widowed	74	6.8
Children		
Yes	697	64.4
No	385	35.6
Place of residence		
rural	343	31.7
city with below 20,000 residents	130	12.0
city with from 20,000 to 99,999 residents	238	22.0
city with from 100,000 to 499,999 residents	207	19.1
city with over 500,000 residents	164	15.2
Educational level		
primary	36	3.3
vocational	93	8.6
secondary	492	45.5
higher	461	42.6
Occupational status		
active	666	61.6
passive	416	38.4
Self-reported financial situation		
good or very good	165	15.3
rather good	307	28.4
moderate/difficult to say	411	38.0
rather bad	97	9.0
bad or very bad	102	9.5

**Table 2:** Respondents’ knowledge regarding the symptoms of head and neck cancers and risk factors for head and neck cancers (*n* = 1082).

	Overall (*n* = 1082)	
Variable	*n*	%
**What do you think are the symptoms of head and neck cancers? (multiple-choice format; positive answers)**		
**Correct answers**		
lump in neck	626	57.9
swelling or lump in the throat	561	51.8
pain or trouble swallowing	425	39.3
chronic sore throat	411	38.0
bleeding in the mouth or throat	410	37.9
chronic hoarseness	408	37.7
numbness of tongue, mouth or lip	373	34.5
red or white sores in the oral cavity that do not heal	316	29.2
ear pain	265	24.5
nasal bleeding	259	23.9
persistent stinging of the tongue	200	18.5
blocked nose on one side	137	12.7
**Incorrect answers**		
headache	539	49.8
dizziness	491	45.4
increase in appetite	20	1.8
**What do you think are the risk factors for head and neck cancers? (multiple-choice format; positive answers)**		
**Correct answers**		
smoking cigarettes/tobacco	683	63.1
excessive alcohol consumption	364	33.6
HPV infection	352	32.5
excessive sunbathing	303	28.0
diet low in fruits	169	15.6
**Incorrect answers**		
tooth decay	280	25.9
smoking marijuana	198	18.3
biting cheeks	129	11.9
eating spicy or fatty foods	123	11.4
frequent talking on cell phone	103	9.5

**Table 3:** Awareness of symptoms of head and neck cancers by sociodemographic factors (*n* = 1082).

	Head and Neck Cancers’ Symptoms—Percentage of Respondents Who Answered “Yes” by Sociodemographic Factors											
Variable	Lump in Neck		Swelling or Lump in the Throat		Pain or Trouble Swallowing		Chronic Sore Throat		Bleeding in the Mouth or Throat		Chronic Hoarseness	
	*n* (%)	*p*	*n* (%)	*p*	*n* (%)	*p*	*n* (%)	*p*	*n* (%)	*p*	*n* (%)	*p*
Gender												
Female (*n* = 569)	352 (61.9%)	0.005	322 (56.6%)	0.001	241 (42.4%)	0.03	232 (40.8%)	0.04	229 (40.2%)	0.09	248 (43.6%)	<0.001
Male (*n* = 513)	274 (53.4%)		239 (46.6%)		184 (35.9%)		179 (34.9%)		181 (35.3%)		160 (31.2%)	
Age (years)												
18–34 (*n* = 350)	215 (61.4%)	0.2	183 (52.3%)	0.6	146 (41.7%)	0.7	138 (39.4%)	0.2	138 (39.4%)	0.7	107 (30.6%)	<0.001
35–49 (*n* = 286)	165 (57.7%)		139 (48.6%)		107 (37.4%)		98 (34.3%)		101 (35.3%)		103 (36.0%)	
50–64 (*n* = 287)	164 (57.1%)		154 (53.7%)		111 (38.7%)		120 (41.8%)		112 (39.0%)		132 (46.0%)	
65+ (*n* = 159)	82 (51.6%)		85 (53.5%)		61 (38.4%)		55 (34.6%)		59 (37.1%)		66 (41.5%)	
Marital status												
single (*n* = 236)	136 (57.6%)	0.005	127 (53.8%)	0.4	100 (42.4%)	0.4	94 (39.8%)	0.1	92 (39.0%)	0.2	84 (35.6%)	0.04
married (*n* = 546)	330 (60.4%)		278 (50.9%)		216 (39.6%)		213 (39.0%)		205 (37.5%)		227 (41.6%)	
informal relationship (*n* = 161)	98 (60.9%)		91 (56.5%)		62 (38.5%)		63 (39.1%)		69 (42.9%)		46 (28.6%)	
divorced (*n* = 65)	34 (52.3%)		32 (49.2%)		25 (38.5%)		24 (36.9%)		24 (36.9%)		26 (40.0%)	
widowed (*n* = 74)	28 (37.8%)		33 (44.6%)		22 (29.7%)		17 (23.0%)		20 (27.0%)		25 (33.8%)	
Children												
Yes (*n* = 697)	400 (57.4%)	0.7	361 (51.8%)	0.9	270 (38.7%)	0.6	266 (38.2%)	0.9	260 (37.3%)	0.6	288 (41.3%)	<0.001
No (*n* = 385)	226 (58.7%)		200 (51.9%)		155 (40.3%)		145 (37.7%)		150 (39.0%)		120 (31.2%)	
Place of residence												
rural (*n* = 343)	196 (57.1%)	0.9	173 (50.4%)	0.8	130 (37.9%)	0.6	118 (34.4%)	0.4	130 (37.9%)	0.9	127 (37.0%)	0.2
city with below 20,000 residents (*n* = 130)	79 (60.8%)		68 (52.3%)		52 (40.0%)		48 (36.9%)		52 (40.0%)		56 (43.1%)	
city with from 20,000 to 99,999 residents (*n* = 238)	137 (57.6%)		124 (52.1%)		86 (36.1%)		90 (37.8%)		86 (36.1%)		80 (33.6%)	
city with from 100,000 to 499,999 residents (*n* = 164)	120 (58.0%)		105 (50.7%)		87 (42.0%)		86 (41.5%)		82 (39.6%)		73 (35.3%)	
city with over 500,000 residents	94 (57.3%)		91 (55.5%)		70 (42.7%)		69 (42.1%)		60 (36.6%)		72 (43.9%)	
Educational level												
primary (*n* = 36)	16 (44.4%)	0.04	13 (36.1%)	0.001	13 (36.1%)	0.3	8 (22.2%)	0.1	12 (33.3%)	0.1	6 (16.7%)	0.01
vocational (*n* = 93)	45 (48.4%)		44 (47.3%)		32 (34.4%)		32 (34.4%)		30 (32.3%)		33 (35.5%)	
secondary (*n* = 492)	284 (57.7%)		235 (47.8%)		184 (37.4%)		184 (37.4%)		174 (35.4%)		176 (35.8%)	
higher (*n* = 461)	281 (61.0%)		269 (58.4%)		196 (42.5%)		187 (40.6%)		194 (42.1%)		193 (41.9%)	
Occupational status												
active (*n* = 666)	393 (59.0%)	0.3	354 (53.2%)	0.3	260 (39.0%)	0.8	251 (37.7%)	0.8	251 (37.7%)	0.9	241 (36.2%)	0.2
passive (*n* = 416)	233 (56.0%)		207 (49.8%)		165 (39.7%)		160 (38.5%)		159 (38.2%)		167 (40.1%)	
Self-reported financial situation												
good or very good (*n* = 165)	101 (61.2%)	0.2	85 (51.5%)	0.1	67 (40.6%)	0.8	65 (39.4%)	0.3	65 (39.4%)	0.2	58 (35.2%)	0.2
rather good (*n* = 307)	192 (62.5%)		177 (57.7%)		126 (41.0%)		130 (42.3%)		130 (42.3%)		128 (41.7%)	
moderate/difficult to say (*n* = 411)	227 (55.2%)		207 (50.4%)		160 (38.9%)		148 (36.0%)		149 (36.3%)		154 (37.5%)	
rather bad (*n* = 97)	51 (52.6%)		43 (44.3%)		34 (35.1%)		34 (35.1%)		29 (29.9%)		28 (28.9%)	
bad or very bad (*n* = 102)	55 (53.9%)		49 (48.0%)		38 (37.3%)		34 (33.3%)		37 (36.3%)		40 (39.2%)	
**Variable**	**Numbness of Tongue, Mouth or Lip**		**Red or White Sores in the Oral Cavity that Do not Heal**		**Ear Pain**		**Nasal Bleeding**		**Persistent Stinging of the Tongue**		**Blocked Nose on One Side**	
	***n* (%)**	***p***	***n* (%)**	***p***	***n* (%)**	***p***	***n* (%)**	***p***	***n* (%)**	***p***	***n* (%)**	***p***
Gender												
Female (*n* = 569)	221 (38.8%)	0.001	180 (31.6%)	0.06	149 (26.2%)	0.2	143 (25.1%)	0.3	122 (21.4%)	0.01	70 (12.3%)	0.7
Male (*n* = 513)	152 (29.6%)		136 (26.5%)		116 (22.6%)		116 (22.6%)		78 (15.2%)		67 (13.1%)	
Age (years)												
18–34 (*n* = 350)	126 (36.0%)	0.1	102 (29.1%)	0.3	92 (26.3%)	0.2	101 (28.9%)	0.03	72 (20.6%)	0.08	50 (14.3%)	0.3
35–49 (*n* = 286)	86 (30.1%)		72 (25.2%)		62 (21.7%)		65 (22.7%)		43 (15.0%)		40 (14.0%)	
50–64 (*n* = 287)	111 (38.7%)		89 (31.0%)		78 (27.2%)		66 (23.0%)		62 (21.6%)		33 (11.5%)	
65+ (*n* = 159)	50 (31.4%)		53 (33.3%)		33 (20.8%)		27 (17.0%)		23 (14.5%)		14 (8.8%)	
Marital status												
single (*n* = 236)	88 (37.3%)	0.7	76 (32.2%)	0.2	63 (26.7%)	0.4	75 (31.8%)	0.001	45 (19.1%)	0.2	39 (16.5%)	0.03
married (*n* = 546)	190 (34.8%)		163 (29.9%)		130 (23.8%		116 (21.2%)		112 (20.5%)		63 (11.5%)	
informal relationship (*n* = 161)	51 (31.7%)		46 (28.6%)		44 (27.3%)		42 (26.1%)		25 (15.5%)		26 (16.1%)	
divorced (*n* = 65)	22 (33.8%)		18 (27.7%)		16 (24.6%)		18 (27.7%)		10 (15.4%)		5 (7.7%)	
widowed (*n* = 74)	22 (29.7%)		13 (17.6%)		12 (16.2%)		8 (10.8%)		8 (10.8%)		4 (5.4%)	
Children												
Yes (*n* = 697)	242 (34.7%)	0.8	209 (30.0%)	0.4	172 (24.7%)	0.8	153 (22.0%)	0.04	132 (18.9%)	0.6	83 (11.9%)	0.3
No (*n* = 385)	131 (34.0%)		107 (27.8%)		93 (24.2%)		106 (27.5%)		68 (17.7%)		54 (14.0%)	
Place of residence												
rural (*n* = 343)	121 (35.3%)	0.8	96 (28.0%)	0.4	79 (23.0%)	0.6	72 (21.0%)	0.4	60 (17.5%)	0.02	41 (12.0%)	0.8
city with below 20,000 residents (*n* = 130)	42 (32.3%)		36 (27.7%)		36 (27.7%)		27 (20.8%)		19 (14.6%)		15 (11.5%)	
city with from 20,000 to 99,999 residents (*n* = 238)	76 (31.9%)		63 (26.5%)		54 (22.7%)		61 (25.6%)		38 (16.0%)		28 (11.8%)	
city with from 100,000 to 499,999 residents (*n* = 164)	74 (35.7%)		64 (30.9%)		50 (24.2%)		54 (26.1%)		55 (26.6%)		29 (14.0%)	
city with over 500,000 residents	60 (36.6%)		57 (34.8%)		46 (28.0%)		45 (27.4%)		28 (17.1%)		24 (14.6%)	
Educational level												
primary (*n* = 36)	9 (25.0%)	0.1	8 (22.2%)	0.1	4 (11.1%)	0.001	7 (19.4%)	0.2	3 (8.3%)	0.01	3 (8.3%)	0.04
vocational (*n* = 93)	23 (24.7%)		21 (22.6%)		19 (20.4%)		15 (16.1%)		14 (15.1%)		7 (7.5%)	
secondary (*n* = 492)	172 (35.0%)		137 (27.8%)		103 (20.9%)		115 (23.4%)		77 (15.7%)		54 (11.0%)	
higher (*n* = 461)	169 (36.7%)		150 (32.5%)		139 (30.2%)		122 (26.5%)		106 (23.0%)		73 (15.8%)	
Occupational status												
active (*n* = 666)	222 (33.3%)	0.3	187 (28.1%)	0.3	183 (27.5%)	0.01	171 (25.7%)	0.09	133 (20.0%)	0.1	97 (14.6%)	0.02
passive (*n* = 416)	151 (36.3%)		129 (31.0%)		82 (19.7%)		88 (21.2%)		67 (16.1%)		40 (9.6%)	
Self-reported financial situation												
good or very good (*n* = 165)	58 (35.2%)	0.4	48 (29.1%)	0.2	45 (27.3%)	0.6	45 (27.3%)	0.2	35 (21.2%)	0.4	18 (10.9%)	0.3
rather good (*n* = 307)	109 (35.5%)		101 (32.9%)		78 (25.4%)		71 (23.1%)		62 (20.2%)		44 (14.3%)	
moderate/difficult to say (*n* = 411)	147 (35.8%)		121 (29.4%)		93 (22.6%)		100 (24.3%)		75 (18.2%)		48 (11.7%)	
rather bad (*n* = 97)	25 (25.8%)		19 (19.6%)		21 (21.6%)		15 (15.5%)		14 (14.4%)		9 (9.3%)	
bad or very bad (*n* = 102)	34 (33.3%)		27 (26.5%)		28 (27.5%)		28 (27.5%)		14 (13.7%)		18 (17.6%)	

**Table 4:** Awareness of risk factors for head and neck cancers by sociodemographic factors (*n* = 1082).

	Risk Factors for Head and Neck Cancers—Percentage of Respondents Who Answered “Yes” by Sociodemographic Factors									
Variable	Excessive Alcohol Consumption		Smoking Cigarettes/Tobacco		HPV Infection		Excessive Sunbathing		Diet Low in Fruits	
	*n* (%)	*p*	*n* (%)	*p*	*n* (%)	*p*	*n* (%)	*p*	*n* (%)	*p*
Gender										
Female (*n* = 569)	194 (34.1%)	0.7	370 (65.0%)	0.2	196 (34.4%)	0.2	163 (28.6%)	0.6	86 (15.1%)	0.6
Male (*n* = 513)	170 (33.1%)		313 (61.0%)		156 (30.4%)		140 (27.3%)		83 (16.2%)	
Age (years)										
18–34 (*n* = 350)	119 (34.0%)	0.9	201 (57.4%)	0.06	110 (31.4%)	0.9	106 (30.3%)	0.6	56 (16.0%)	0.1
35–49 (*n* = 286)	98 (34.3%)		189 (66.1%)		96 (33.6%)		72 (25.2%)		36 (12.6%)	
50–64 (*n* = 287)	92 (32.1%)		186 (64.8%)		94 (32.8%)		81 (28.2%)		43 (15.0%)	
65+ (*n* = 159)	55 (34.6%)		107 (67.3%)		52 (32.7%)		44 (27.7%)		34 (21.4%)	
Marital status										
single (*n* = 236)	88 (37.3%)	0.6	140 (59.3%)	0.03	69 (29.2%)	0.6	71 (30.1%)	0.03	38 (16.1%)	0.2
married (*n* = 546)	174 (31.9%)		356 (65.2%)		186 (34.1%)		161 (29.5%)		92 (16.8%)	
informal relationship (*n* = 161)	60 (37.3%)		106 (65.8%)		55 (34.2%)		46 (28.6%)		20 (12.4%)	
divorced (*n* = 65)	26 (40.0%)		45 (69.2%)		22 (33.8%)		16 (24.6%)		13 (20.0%)	
widowed (*n* = 74)	16 (21.6%)		36 (48.6%)		20 (27.0%)		9 (12.2%)		6 (8.1%)	
Children										
Yes (*n* = 697)	232 (33.3%)	0.7	453 (65.0%)	0.09	237 (34.0%)	0.2	190 (27.3%)	0.5	109 (15.6%)	0.9
No (*n* = 385)	132 (34.3%)		230 (59.7%)		115 (29.9%)		113 (29.4%)		60 (15.6%)	
Place of residence										
rural (*n* = 343)	106 (30.9%)	0.2	207 (60.3%)	0.4	111 (32.4%)	0.05	102 (29.7%)	0.7	62 (18.1%)	0.6
city with below 20,000 residents (*n* = 130)	41 (31.5%)		81 (62.3%)		39 (30.0%)		34 (26.2%)		17 (13.1%)	
city with from 20,000 to 99,999 residents (*n* = 238)	77 (32.4%)		147 (61.8%)		63 (26.5%)		59 (24.8%)		34 (14.3%)	
city with from 100,000 to 499,999 residents (*n* = 164)	83 (40.1%)		136 (65.7%)		82 (39.6%)		62 (30.0%)		29 (14.0%)	
city with over 500,000 residents	57 (34.8%)		112 (68.3%)		57 (34.8%)		46 (28.0%)		27 (16.5%)	
Educational level										
primary (*n* = 36)	11 (30.6%)	0.01	20 (55.6%)	0.04	7 (19.4%)	0.04	2 (5.6%)	0.02	5 (13.9%)	0.9
vocational (*n* = 93)	20 (21.5%)		50 (53.8%)		25 (26.9%)		25 (26.9%)		13 (14.0%)	
secondary (*n* = 492)	158 (32.1%)		303 (61.6%)		151 (30.7%)		138 (28.0%)		75 (15.2%)	
higher (*n* = 461)	175 (38.0%)		310 (67.2%)		169 (36.7%)		138 (29.9%)		76 (16.5%)	
Occupational status										
active (*n* = 666)	231 (34.7%)	0.4	413 (62.0%)	0.3	229 (34.4%)	0.1	187 (28.1%)	0.9	100 (15.0%)	0.5
passive (*n* = 416)	133 (32.0%)		270 (64.9%)		123 (29.6%)		116 (27.9%)		69 (16.6%)	
Self-reported financial situation										
good or very good (*n* = 165)	60 (36.4%)	0.1	106 (64.2%)	0.06	50 (30.3%)	0.3	57 (34.5%)	0.08	25 (15.2%)	0.8
rather good (*n* = 307)	115 (37.5%)		209 (68.1%)		114 (37.1%)		89 (29.0%)		47 (15.3%)	
moderate/difficult to say (*n* = 411)	136 (33.1%)		248 (60.3%)		123 (29.9%)		111 (27.0%)		63 (15.3%)	
rather bad (*n* = 97)	25 (25.8%)		65 (67.0%)		29 (29.9%)		27 (27.8%)		14 (14.4%)	
bad or very bad (*n* = 102)	28 (27.5%)		55 (53.9%)		36 (35.5%)		19 (18.6%)		20 (19.6%)	

**Table 5:** Factors associated with awareness of selected symptoms of head and neck cancers: Odds ratios (OR) and 95% confidence intervals (95%CI), *n* = 1082.

	Factors Associated with Awareness of Selected Symptoms of Head and Neck Cancers									
Variable	Lump in Neck		Swelling or Lump in the Throat		Chronic Hoarseness		Numbness of Tongue, Mouth or Lip		Blocked Nose on One Side	
	Univariate Logistic Regression	Multivariate Logistic Regression	Univariate Logistic Regression	Multivariate Logistic Regression	Univariate Logistic Regression	Multivariate Logistic Regression	Univariate Logistic Regression	Multivariate Logistic Regression	Univariate Logistic Regression	Multivariate Logistic Regression
Overall	OR (95%CI)	OR (95%CI)	OR (95%CI)	OR (95%CI)	OR (95%CI)	OR (95%CI)	OR (95%CI)	OR (95%CI)	OR (95%CI)	OR (95%CI)
Gender										
Female	1.42 (1.11–1.80) **	1.43 (1.11–1.83) **	1.50 (1.18–1.90) **	1.56 (1.22–2.00) ***	1.71 (1.33–2.19) ***	1.66 (1.28–2.14) ***	1.51 (1.17–1.94) **	1.46 (1.13–1.90) **	0.93 (0.65–1.34)	0.97 (0.67–1.40)
Male	Reference	Reference	Reference	Reference	Reference	Reference	Reference	Reference	Reference	Reference
Age (years)										
18–34	1.50 (1.03–2.18) *	1.45 (0.90–2.33)	0.95 (0.66–1.39)	0.66 (0.41–1.07)	0.62 (0.42–0.92) *	0.68 (0.42–1.11)	1.23 (0.82–1.83)	1.29 (0.79–2.12)	1.73 (0.92–3.23)	1.17 (0.54–2.53)
35–49	1.28 (0.87–1.89)	1.24 (0.77–1.99)	0.82 (0.56–1.21)	0.59 (0.37–0.96) *	0.79 (0.53–1.18)	0.83 (0.51–1.36)	0.94 (0.62–1.43)	1.02 (0.62–1.68)	1.68 (0.89–3.20)	1.18 (0.55–2.53)
50–64	1.25 (0.85–1.85)	1.20 (0.78–1.85)	1.01 (0.68–1.49)	0.83 (0.54–1.28)	1.20 (0.81–1.78)	1.22 (0.78–1.89)	1.38 (0.91–2.07)	1.43 (0.91–2.26)	1.35 (0.70–2.60)	1.12 (0.54–2.32)
65+	Reference	Reference	Reference	Reference	Reference	Reference	Reference	Reference	Reference	Reference
Marital status										
ever married	Reference	Reference	Reference	Reference	Reference	Reference	Reference	Reference	Reference	Reference
never married	1.07 (0.84–1.38)	1.01 (0.71–1.43)	1.21 (0.95–1.56)	1.59 (1.11–2.27) *	0.71 (0.55–92) *	1.12 (0.78–1.61)	1.04 (0.80–1.35)	1.17 (0.81–1.69)	1.67 (1.16–2.39) **	1.89 (1.15–3.10) *
Children										
Yes	0.95 (0.74–1.22)	1.00 (0.70–1.42)	0.99 (0.78–1.28)	1.17 (0.82–1.66)	1.56 (1.20–2.02) **	1.33 (0.93–1.92)	1.03 (0.79–1.34)	1.13 (0.78–1.63)	0.83 (0.57–1.20)	1.32 (0.80–2.20)
No	Reference	Reference	Reference	Reference	Reference	Reference	Reference	Reference	Reference	Reference
Place of residence										
rural	0.99 (0.68–1.45)	1.00 (0.68–1.49)	0.82 (0.56–1.19)	1.00 (0.68–1.48)	0.75 (0.52–1.10)	0.91 (0.61–1.35)	0.95 (0.64–1.39)	1.01 (0.67–1.51)	0.79 (0.46–1.36)	0.85 (0.48–1.49)
city with below 20,000 residents	1.15 (0.72–1.84)	1.16 (0.72–1.87)	0.88 (0.55–1.40)	1.00 (0.62–1.61)	0.97 (0.61–1.54)	1.11 (0.69–1.80)	0.83 (0.51–1.35)	0.86 (0.52–1.42)	0.76 (0.38–1.52)	0.78 (0.38–1.58)
city with from 20,000 to 99,999 residents	1.01 (0.68–1.51)	1.01 (0.67–1.53)	0.87 (0.59–1.30)	0.92 (0.61–1.38)	0.65 (0.43–0.97) *	0.66 (0.43–0.99) *	0.81 (0.54–1.24)	0.81 (0.53–1.24)	0.78 (0.43–1.40)	0.81 (0.45–1.48)
city from 100,000 to 499,999 with residents	1.03 (0.68–1.56)	1.01 (0.67–1.55)	0.83 (0.55–1.25)	0.87 (0.57–1.32)	0.70 (0.46–1.06)	0.72 (0.46–1.10)	0.96 (0.63–1.48)	0.95 (0.62–1.47)	0.95 (0.53–1.71)	0.94 (0.52–1.71)
city with over 500,000 residents	Reference	Reference	Reference	Reference	Reference	Reference	Reference	Reference	Reference	Reference
Higher education										
yes	1.25 (0.98–1.60)	1.22 (0.95–1.58)	1.58 (1.24–2.01) ***	1.57 (1.22–2.03) ***	1.36 (1.06–1.74) *	1.42 (1.09–1.84) **	1.18 (0.92–1.52)	1.24 (0.95–1.61)	1.64 (1.14–2.35) **	1.59 (1.09–2.30) *
no	Reference	Reference	Reference	Reference	Reference	Reference	Reference	Reference	Reference	Reference
Occupational status										
active	1.13 (0.88–1.45)	1.00 (0.74–1.35)	1.15 (0.90–1.46)	1.24 (0.92–1.67)	0.85 (0.66–1.09)	0.87 (0.64–1.18)	0.88 (0.70–1.13)	0.81 (0.60–1.10)	1.60 (1.08–2.37) *	1.39 (0.87–2.20)
passive	Reference	Reference	Reference	Reference	Reference	Reference	Reference	Reference	Reference	Reference
Self-reported financial situation										
rather good, good or very good	1.44 (1.03–2.01) *	1.36 (0.68–1.49)	1.45 (1.04–2.02) *	1.38 (0.98–1.95)	1.25 (0.89–1.77)	1.32 (0.92–1.89)	1.30 (0.91–1.86)	1.32 (0.91–1.90)	0.96 (0.59–1.57)	0.86 (0.52–1.42)
moderate/difficult to say	1.08 (0.77–1.52)	1.04 (0.74–1.47)	1.18 (0.84–1.66)	1.17 (0.82–1.65)	1.15 (0.81–1.65)	1.23 (0.85–1.78)	1.32 (0.92–1.90)	1.36 (0.93–1.97)	0.84 (0.51–1.40)	0.80 (0.48–1.34)
rather bad, bad or very bad	Reference	Reference	Reference	Reference	Reference	Reference	Reference	Reference	Reference	Reference

* *p* < 0.05; ** *p* < 0.01; *** *p* < 0.001.

**Table 6:** Factors associated with awareness of risk factors for head and neck cancers: Odds ratios (OR) and 95% confidence intervals (95%CI), *n* = 1082.

	Factors Associated with Awareness of Risk Factors for Head and Neck Cancers									
Variable	Excessive Alcohol Consumption		Smoking Cigarettes/Tobacco		HPV Infection		Excessive Sunbathing		Diet Low in Fruits	
	Univariate Logistic Regression	Multivariate Logistic Regression	Univariate Logistic Regression	Multivariate Logistic Regression	Univariate Logistic Regression	Multivariate Logistic Regression	Univariate Logistic Regression	Multivariate Logistic Regression	Univariate Logistic Regression	Multivariate Logistic Regression
Overall	OR (95%CI)	OR (95%CI)	OR (95%CI)	OR (95%CI)	OR (95%CI)	OR (95%CI)	OR (95%CI)	OR (95%CI)	OR (95%CI)	OR (95%CI)
Gender										
Female	1.04 (0.81–1.34)	1.06 (0.82–1.38)	1.19 (0.93–1.52)	1.17 (0.90–1.51)	1.20 (0.93–1.55)	1.20 (0.92–1.56)	1.07 (0.82–1.40)	1.08 (0.82–1.42)	0.92 (0.66–1.28)	0.94 (0.67–1.32)
Male	Reference	Reference	Reference	Reference	Reference	Reference	Reference	Reference	Reference	Reference
Age (years)										
18–34	0.97 (0.66–1.45)	0.76 (0.46–1.26)	0.66 (0.44–0.97) *	0.71 (0.43–1.16)	0.94 (0.63–1.41)	0.82 (0.49–1.36)	1.14 (0.75–1.72)	0.98 (0.58–1.65)	0.70 (0.44–1.13)	0.63 (0.34–1.17)
35–49	0.99 (0.66–1.48)	0.82 (0.49–1.34)	0.95 (0.63–1.43)	1.05 (0.64–1.72)	1.04 (0.69–1.57)	0.81 (0.49–1.34)	0.88 (0.57–1.36)	0.78 (0.46–1.32)	0.53 (0.32–0.89) *	0.44 (0.24–0.84) *
50–64	0.89 (0.59–1.34)	0.80 (0.51–1.27)	0.90 (0.59–1.35)	0.97 (0.61–1.52)	1.00 (0.66–1.52)	0.82 (0.52–1.31)	1.03 (0.67–1.58)	0.97 (0.60–1.57)	0.65 (0.39–1.07)	0.60 (0.34–1.06)
65+	Reference	Reference	Reference	Reference	Reference	Reference	Reference	Reference	Reference	Reference
Marital status										
ever married	Reference	Reference	Reference	Reference	Reference	Reference	Reference	Reference	Reference	Reference
never married	1.29 (0.99–1.67)	1.57 (1.09–2.26) *	0.93 (0.72–1.19)	1.29 (0.90–1.86)	0.91 (0.70–1.19)	1.07 (0.74–1.54)	1.12 (0.85–1.47)	1.11 (0.76–1.63)	0.86 (0.63–1.25)	0.86 (0.53–1.40)
Children										
Yes	0.96 (0.74–1.24)	1.23 (0.85–1.78)	1.25 (0.97–1.62)	1.27 (0.89–1.82)	1.21 (0.93–1.58)	1.24 (0.86–1.79)	0.90 (0.69–1.19)	0.99 (0.67–1.45)	1.00 (0.71–1.42)	0.90 (0.56–1.45)
No	Reference	Reference	Reference	Reference	Reference	Reference	Reference	Reference	Reference	Reference
Place of residence										
rural	0.84 (0.57–1.25)	0.92 (0.61–1.38)	0.71 (0.48–1.05)	0.79 (0.52–1.18)	0.90 (0.61–1.33)	0.99 (0.66–1.49)	1.09 (0.72–1.64)	1.16 (0.75–1.77)	1.12 (0.68–1.84)	1.29 (0.77–2.17)
city with below 20,000 residents	0.87 (0.53–1.41)	0.89 (0.54–1.47)	0.77 (0.47–1.25)	0.82 (0.50–1.34)	0.81 (0.49–1.32)	0.84 (0.51–1.39)	0.91 (0.54–1.53)	0.93 (0.55–1.58)	0.76 (0.40–1.47)	0.84 (0.43–1.63)
city with from 20,000 to 99,999 residents	0.90 (0.59–1.37)	0.90 (0.58–1.38)	0.75 (0.49–1.14)	0.76 (0.50–1.17)	0.68 (0.44–1.04)	0.69 (0.45–1.07)	0.85 (0.54–1.33)	0.85 (0.54–1.34)	0.85 (0.49–1.47)	0.89 (0.51–1.56)
city with from 100,000 to 499,999 residents	1.26 (0.82–1.92)	1.29 (0.84–1.99)	0.89 (0.58–1.38)	0.92 (0.60–1.44)	1.23 (0.81–1.88)	1.27 (0.82–1.95)	1.10 (0.70–1.72)	1.12 (0.71–1.77)	0.83 (0.47–1.46)	0.90 (0.50–1.60)
city with over 500,000 residents	Reference	Reference	Reference	Reference	Reference	Reference	Reference	Reference	Reference	Reference
Higher education										
yes	1.40 (1.09–1.80) **	1.33 (1.02–1.73) *	1.37 (1.06–1.76) *	1.32 (1.01–1.71) *	1.39 (1.07–1.79) *	1.32 (1.01–1.73) *	1.18 (0.90–1.54)	1.18 (0.90–1.56)	1.12 (0.81–1.56)	1.17 (0.83–1.65)
no	Reference	Reference	Reference	Reference	Reference	Reference	Reference	Reference	Reference	Reference
Occupational status										
active	1.13 (0.87–1.47)	1.10 (0.80–1.50)	0.88 (0.68–1.14)	0.85 (0.63–1.15)	1.25 (0.96–1.63)	1.30 (0.94–1.79)	1.01 (0.77–1.33)	1.01 (0.73–1.40)	0.89 (0.64–1.24)	1.15 (0.76–1.75)
passive	Reference	Reference	Reference	Reference	Reference	Reference	Reference	Reference	Reference	Reference
Self-reported financial situation										
rather good, good or very good	1.62 (1.13–2.34) **	1.60 (1.10–2.33) *	1.32 (0.94–1.86)	1.38 (0.97–1.96)	1.10 (0.77–1.56)	1.07 (0.74–1.53)	1.49 (1.02–2.19) *	1.49 (1.01–2.20) *	0.87 (0.56–1.37)	0.88 (0.56–1.38)
moderate/difficult to say	1.36 (0.94–1.98)	1.37 (0.94–2.01)	1.00 (0.71–1.42)	1.06 (0.74–1.50)	0.88 (0.61–1.27)	0.87 (0.60–1.27)	1.23 (0.83–1.83)	1.24 (0.83–1.85)	0.88 (0.56–1.39)	0.89 (0.56–1.41)
rather bad, bad or very bad	Reference	Reference	Reference	Reference	Reference	Reference	Reference	Reference	Reference	Reference

* *p* < 0.05; ** *p* < 0.01.

## Data Availability

Data are available on reasonable request. The dataset used to conduct the analyses is available from the corresponding author on reasonable request.
